# Editorial: Functional Profile of the Lipocalin Protein Family

**DOI:** 10.3389/fphys.2022.904702

**Published:** 2022-04-28

**Authors:** Maria Dolores Ganfornina, Bo Åkerström, Diego Sanchez

**Affiliations:** ^1^ Instituto de Biologia y Genetica Molecular, Unidad de Excelencia, Universidad de Valladolid-Consejo Superior de Investigaciones Cientificas, Valladolid, Spain; ^2^ Department of Clinical Sciences in Lund, Lund University, Lund, Sweden

**Keywords:** immunomodulation, antioxidant, cellular barriers, metabolism, reproduction, development, social behaviour, moonlighting proteins

## Introduction

Lipocalins form an ancestral protein family found in all kingdoms of life, except for Archaea. From a small number of family members, Lipocalins rapidly evolved through duplication and divergence in the vertebrate genome, giving rise to nineteen different proteins in humans. Despite a high sequence diversity of homologous Lipocalins, their tertiary structure displays a resilient fold of eight beta-barrels delimiting a binding pocket where they can accommodate different ligands, mostly hydrophobic.

A close-to-linear accrual of publications marks the knowledge accumulation on Lipocalins since the family name was coined in 1985, but an inflection point appears in the literature after 2006, when the last comprehensive review of this protein family was collected. Since then, an explosion of association studies of Lipocalin expression with many human diseases has taken place, ranging from metabolic and endocrine syndromes to cancer, cardiovascular, neurodegenerative and psychiatric conditions. Regardless of this wealth of correlational data, with their understandable practical use as disease biomarkers, an analysis of publications devoted to Lipocalin biological function was needed.

**FIGURE 1:**
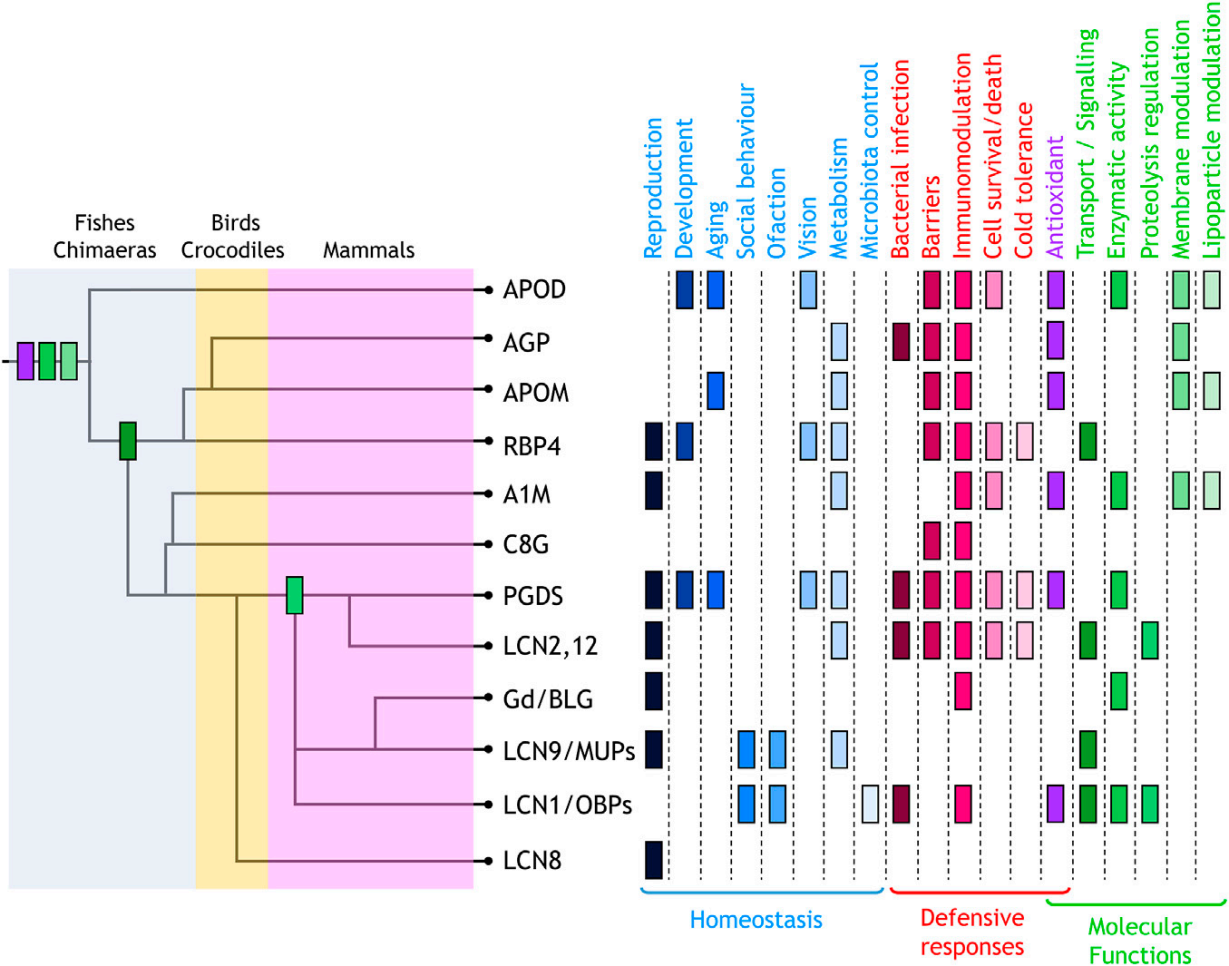
Lipocalin functions mapped on the dendrogram of Lipocalin evolutionary history (Diez-Hermano et al.) where major monophyletic clades are shown. Physiological functions identified are classified into homeostatic (blue tones) and defensive (red tones). Functions defined at the molecular level (green tones) also include antioxidant (purple). The first appearance of these molecular functions in the evolutionary path of chordate Lipocalin are indicated in the tree using the same colour code. Antioxidant, enzymatic activity, and membrane modulation are ancestral functions. The current knowledge places transport/signalling at the divergence of RBP4 and PGDS-related radiation. In contrast, proteolysis regulation appeared later in evolution.

In this Research Topic Issue, 28 authors have contributed a valuable collection of eleven reviews, focused on the function of most vertebrate Lipocalins. Their analyses, at every organizational level (molecular, cellular, tissues or organ systems), uncover an interesting pattern where the common Lipocalin structure provides a basic biochemical tool put to work in an amazingly varied set of physiological and pathological contexts. Specializations are combined with shared properties, and labour division with functional redundancy. The Research Topic starts with a novel view of Lipocalins evolution in chordates (Diez-Hermano et al.), where the use of unbiased selection of animal species, have yielded a phylogenetic tree with strong support of previously elusive relationships. On this evolutionary pattern, we can now map the Lipocalin functions (Figure 1), updated by all contributors to the Topic.

Two major categories can be established: homeostatic functions, regulating life processes like reproduction, development, or aging, and defensive functions triggered in response to damage or disease. Behind these physiological roles lie molecular functions that exploit the ligand binding pocket, the surface for intermolecular interactions, and a set of residues involved in enzymatic activities.

A rampant plethora of names has appeared for the individual Lipocalins, especially during the early years. Although several attempts to systematization of Lipocalin nomenclature have failed, a consensus in the name usage for each Lipocalin has slowly been reached during recent years. In this Editorial and Figure we have settled upon the names, abbreviations and acronyms used in the separate contributions in the Research Topic Issue.

## The Ligand Partners of Lipocalins

The family name was adequately chosen to highlight a prominent feature of Lipocalins: the pocket within the calix that can bind ligands. Ligand binding properties can be highly specific [e.g., retinol transport by RBP4 (Steinhoff et al.)], or rather “promiscuous” [AGP or Lcn1 being capable of binding many endogenous or exogenous molecules (Glasgow; Ruiz)].

Ligand binding can fulfil different purposes and have different spatial scales. Transport of ligands to target tissues to perform a function [RBP transport in the retina or Lcn2 as iron-provider (Bhusal et al.; Steinhoff et al.)] or the removal of toxic molecules [scavenging roles of Lcn1, OBPs or A1M (Bergwik et al.; Glasgow; Penn et al.)] are genuine transporter functions of Lipocalins. However, ligand-managing is not reduced to transport; Lipocalins can use the ligand to transform it [diverse enzymatic activities evolved in different family members like ApoD, A1M, PGDS or Lcn1 (Bergwik et al.; Glasgow; Sanchez and Ganfornina; Urade)], to prevent its oxidation [A1M heme quenching (Bergwik et al.)], or to retain a ligand in intra- or extracellular fluids [e.g., MUPs maintenance of pheromones in urinary scent marks guiding sexual and social behaviour (Penn et al.)]. Moreover, the ability of abundant Lipocalins to bind xenobiotics (including pharmacological agents) must be considered when analysing the pharmacokinetics and expected outcome of a treatment (Ruiz).

Biochemical moonlighting is also clear for some Lipocalins, where enzymatic activities (Bergwik et al.; Sanchez and Ganfornina; Urade) carried out by Met93 in ApoD, Cys34 in A1M or Cys65 in PGDS (all curiously located close to the binding pocket rim) can coexist with binding ligands deeper in the pocket, with physiological roles that can be experimentally dissociated.

As relevant as ligand-binding in the pocket can be for a Lipocalin, important functions have been demonstrated that employ interactions with high molecular weight partners, such as protein-protein interactions, or are unrelated to the ligand. Examples are the ligand-independent activation of TLR2/4 signalling in immune system cells by RBP4 (Steinhoff et al.), heparin binding by A1M (Bergwik et al.), or the nutritive and protective role of BLG [providing amino acids, and antioxidant and antibacterial peptides to the progeny (Sawyer)]. Indeed, surface residues potentially involved in ligand-independent functions are found as traces in the evolution of the different Lipocalin clades (Diez-Hermano et al.) paralleling their large functional divergence.

Finally, most Lipocalins are glycosylated and the oligosaccharides provide the basis of functional interactions of the Lipocalin, like the lectin-like binding of Gd to cell surfaces of endometrial decidua, oocyte and spermatozoa (Sawyer). Also, variation in glycosylation patterns are observed in various Lipocalins that can condition their interactions [ApoD, AGP, Gd (Ruiz; Sanchez and Ganfornina; Sawyer)] and are worth further analysis.

## Lipocalins in the Battlefield: Immune and Defensive Responses

A prominent function of Lipocalins is their role as acute response proteins upon harmful stimuli (infection, inflammation, injury or disease) where up-regulation of Lipocalin expression is part of the general response. Lipocalins can synthesize immune system mediators [PGD_2_ (Urade)] or control the availability of small immunomodulating molecules [like LPC by ApoD, or LPC and PAF by AGP (Ruiz; Sanchez and Ganfornina)]. They can either promote inflammation [PGDS or Lcn2 (Bhusal et al.; Urade)], restrain its extent or duration [ApoD, AGP (Ruiz; Sanchez and Ganfornina)], or have immunosuppressive functions [Gd, A1M (Bergwik et al.; Sawyer)]. They can control bacterial infections by scavenging precious iron-containing siderophores [AGP, Lcn2, C8G, Lcn1 and OBPs (Ruiz; Glasgow; Bhusal et al.; Stopková et al.)], transport lipids with antibacterial or antiviral activity or even degrade microbial DNA [Lcn1 (Glasgow)]. This general defensive function is part of the ancestral toolkit of Lipocalins (Figure 1) and lead to the concepts of Immunocalins or Siderocalins, applied to various members of the family [AGP, Lcn2, C8G, Lcn1, OBPs (Bhusal et al.; Glasgow; Ruiz; Stopková et al.)].

This Research Topic highlights yet another function also predicted to be ancestral in the family: regulation of barriers, so important for biological compartments protection [e.g., regulation of endothelial barriers to the passage of immune system cells by AGP (Ruiz), Gd or ApoM (Frances et al., 2021; Yao Mattisson and Christoffersen, 2021; Penn et al.) or the antagonistic roles of C8G and Lcn2 in preventing or promoting blood-brain-barrier disruption (Frances et al., 2021; Kim et al., 2021)]. Furthermore, many Lipocalins share functions dealing with the maintenance, composition and redox state of the most basic barriers of all: cellular membranes, with special attention to plasma and lysosomal membranes [ApoD, AGP, A1M, Gd (Bergwik et al.; Ruiz; Sanchez and Ganfornina; Sawyer)] vital for cell survival-death fates.

## Lipocalins as Guardians of Oxidation State

It has become clear that antioxidant functions are also shared properties of Lipocalins (Figure 1) and they can be achieved by different molecular mechanisms: 1) by quenching oxidable molecules [PGDS, A1M (Bergwik et al.; Urade)]; 2) by scavenging oxidized molecules or radicals that propagate oxidation [OBPs, A1M (Bergwik et al.; Stopková et al.)]; 3) or by direct antioxidant activity, like Met-based lipid reduction by ApoD or Cys-based reduction by A1M (Bergwik et al.; Sanchez and Ganfornina).

Antioxidant effects of Lipocalins can be exerted on small free ligands (A1M), or on higher order lipidic structures by direct interaction with membranes (ApoD, AGP) or lipoprotein particles (ApoD, ApoM) (Bergwik et al.; Ruiz; Sanchez and Ganfornina; Christoffersen and Nielsen, 2013).

## Lipocalins for Hungry Animals in a Chilly World

As for other lipid-binding proteins, metabolism regulation has been assigned to practically all Lipocalins, and correlations of metabolic diseases with Lipocalin expression are abundant. While the causal chain in some metabolic alterations observed upon modifying Lipocalin expression in model organisms still needs to be elucidated, examples of direct regulation of important metabolism-controlling signalling pathways have been demonstrated, like the regulation of insulin pathway by RBP or AGP (Ruiz; Steinhoff et al.) and the involvement in ER-stress, fat deposition and obesity of A1M (Bergwik et al., 2021b). Also, Lcn2 and AGP exert a direct control of food intake behaviours (Bhusal et al.; Ruiz). Metabolic response to cold stress is shared by RBP4, PGDS and Lcn2 (Bhusal et al.; Steinhoff et al.; Urade), a trait curiously present in the distant plant lipocalins (Frenette Charron et al., 2005). Indirect effects on metabolism are also accomplished by the modulation of lipoprotein composition and dynamics [ApoD (Sanchez and Ganfornina), ApoM (Frej et al., 2017)].

## Lipocalins and Sex. Reproduction and Social Behaviour

Lipocalins also play key roles in reproductive organ development and function, as well as in behaviours conditioning reproductive success. They influence various physiological processes like development or maintenance of reproductive organs [Lcn8 or PGDS (Stopková et al.; Urade)], prevention of oxidative stress in placenta [A1M (Bergwik et al.)], nutritive and protecting properties of milk [BLG (Sawyer)], fertilization, implantation and endometrial homeostasis [Gd (Sawyer)], or long-range sex-specific signal communication by MUPs and OBPs (Stopková et al.; Penn et al.). Indeed, the control of microbiota by OBPs not only influence host physiology, but also the perception of health state by putative mates (Stopková et al.).

## Lipocalins and Life. Development and Aging

The well-known roles of retinoic acid in embryonic development place RBP4 as a major player in developmental processes (Steinhoff et al.), but other Lipocalins cannot be discarded due to the shared ability of binding retinoids. Specific lipocalin-regulated developmental processes include development of organ-blood barriers in female and male reproductive organs [functionally linked to retinoic acid, and mediated by RBP receptor STRA6 (Steinhoff et al.)], but also unrelated mechanisms like angiogenesis or chondrogenesis [ApoD (Sanchez and Ganfornina)], or myelin development and maintenance [ApoD and PGDS (Sanchez and Ganfornina; Urade)]. Also, various Lipocalins have roles in the functional maintenance of specific organs upon aging [ApoD, ApoM and PGDS, taking care of brain (Sanchez and Ganfornina), liver (Ding et al., 2020) or cartilage aging (Urade)].

## Where do Lipocalins Functions Take Place?

All vertebrate Lipocalins have a signal peptide that, except for ApoM (Yao Mattisson and Christoffersen, 2021), is cleaved when the polypeptide is synthesized in the RER. There, they begin their path to the extracellular milieu through canonical secretion. Extracellular location is therefore shared by all Lipocalins. However, the picture is getting complicated since various Lipocalins are known to internalize in cells, and traffic to lysosomes either to be degraded [RBP or PGDS (Steinhoff et al.; Urade)] or to perform their function there [ApoD (Sanchez and Ganfornina)]. They can also bind to mitochondria in damaged cells [A1M (Bergwik et al.)].

Furthermore, extracellular Lipocalins appear in different formats: bound to protein partners [RBP4-transthyretin complex (Steinhoff et al.); A1M-IgA complex (Bergwik et al.)], lipoprotein particles [ApoD (Sanchez and Ganfornina), ApoM (Christoffersen and Nielsen, 2013)] or exosomes [ApoD (Sanchez and Ganfornina)]. Lipocalin dynamics within extracellular fluids or intracellular organelles have an important dependence not only on the format used, but also on their glycosylation “coat” that conditions their interactions, stability against proteases, or renal clearance (Ruiz; Sanchez and Ganfornina; Sawyer).

## Are Lipocalins Essential for Life?

Not a single Lipocalin-KO animal model results in lethality, but no null-mutation for a Lipocalin is reported in humans. This paradox can be solved by three Lipocalins facts: 1) Functional redundancy: several Lipocalins can cooperate in the same physiological process [e.g., AGP-RBP4, Lcn2-AGP, RBP4-PGDS (Bhusal et al.; Ruiz; Steinhoff et al.; Urade)]. 2) Labour division: similar functions are performed by different lipocalins in different body compartments [e.g., siderophore scavenging by Lcn1 in tears and Lcn2 in plasma (Bhusal et al.; Glasgow)]. 3) Functional convergence: the enzymatic activity of PGDS is shared by the hematopoietic PGD_2_-synthase (Urade).

## Lipocalins as Optimizers

A major conclusion we extract from the wide array of physiological processes reviewed in this Research Topic is that Lipocalins are key to organismal fitness, despite not being indispensable genes. Instead, they function as optimizers of several processes essential for life.

We need more experimental work to determine if loss of more than one Lipocalin is lethal (synthetic lethality) or if they are conditionally essential in particular environmental or internal stressful situations. Nevertheless, we can conclude that the proper response of an organism to disease or damage clearly depends on optimal performance of Lipocalins.

The functional map collaboratively built from this Research Topic can be a good starting point guiding future research on previously unpredicted functional properties of particular Lipocalins. It should help to increase our knowledge on important physiological processes and expand the applicability of Lipocalins to improve therapies for many pathological conditions.
